# 

**Published:** 1907-06-15

**Authors:** 


THE HOSPITAL June 15, 1907.

*Name* ..........................................................................

*Address* ......................................................................

..........................................................................

This Coupon must accompany manuscript or contributions intended for The Hospital.

